# Neuroimaging insights into transgender and gender nonconfirming youth: a scoping review

**DOI:** 10.1186/s13034-025-00987-1

**Published:** 2025-11-26

**Authors:** Linda M. Bonnekoh, Angela Rölver, Ida Wessing, Ruth H. Fellmeth, Navid Schürmeyer, Udo Dannlowski, Georg Romer

**Affiliations:** 1 Department of Child and Adolescent Psychiatry, Psychosomatics and Psychotherapy, University of Münster, Schmeddingstraße 50, 48149 Münster, Germany; 2 Institute for Translational Psychiatry, University of Münster, Münster, Germany; 3 Department of Psychiatry, Protestant Hospital of the Bethel Foundation Medical School and University Medical Center OWL, Bielefeld University, Bielefeld, Germany

**Keywords:** Adolescents, Young adults, Gender incongruence, Gender dysphoria, Transgender, Non-binary, MRI, Neuroimaging

## Abstract

**Background:**

Gender incongruence in adolescents is a relevant condition in which the gender identity of a person does not correspond with the birth-assigned sex. Individuals with gender dysphoria can experience a distressing burden. The etiology seems to be predominantly biological, but only a few neurobiological correlates have been identified and are not yet embedded in a broader context. The aim of this review is to present an overview about the current state of the literature regarding MRI brain findings in adolescents < 22 years of age with GD/GI also including non-binary identifying adolescents < 22 years. Findings will be discussed considering (a) sample characteristics, (b) the status of possible hormonal therapy including puberty blockers and (c) non-binary identifying persons and from a general ethical perspective.

**Methods:**

A systematic review was conducted using PubMed/Medline with keywords: “gender dysphoria,” “gender incongruence,” “gender identity,” “non-binary,” “MRI,” and “adolescents.” The term “transsexualism” was used to include literature published before the term change related to ICD-11. The protocol was pre-registered (INPLASY2023110074).

**Results:**

We scanned 360 articles and finally included n = 20 studies in the review. We found that studies on adolescents with gender dysphoria are mostly addressing functional imaging. Studies were predominantly limited by cross-sectional designs. N = 3 longitudinal studies were performed in female-to-male-transgender persons before and after beginning with gender affirming hormonal therapy (GAHT). To our knowledge there has never been performed a longitudinal study in male to female adolescents during GAHT. Non-binary aspects were mostly not taken into account. Ethical implications concerning the interpretation of neuroimaging findings in this field have been rarely discussed.

**Conclusion:**

There is a lack of robust neuroimaging data on GI/GD adolescents, particularly from longitudinal studies, which are crucial for understanding neurobiological processes. Non-binary identifying adolescents should be given more consideration in research. Future research should include non-binary adolescents and integrate ethical considerations to prevent misinterpretation or stigmatization. Neuroimaging should not reduce gender diversity to neurobiology but rather contribute to a more nuanced understanding of gender identity.

**Supplementary Information:**

The online version contains supplementary material available at 10.1186/s13034-025-00987-1.

## Background

Gender incongruence (GI) is a relevant condition in which the gender identity of a person does not correspond with the sex assigned at birth [1]. According to Diagnostic and Statistical Manual of Mental Disorders (DSM-5) criteria, gender dysphoria (GD) can be understood as persisting distress resulting from incongruence between birth-assigned sex and the gender the individual identifies with [2].

In recent years, the number of adolescents and young adults seeking treatment for gender incongruence and gender dysphoria has risen steadily in the healthcare system [3, 4]. Furthermore, the total number of assigned diagnoses have increased along with a decrease in the mean age of diagnosis [5] affecting especially health services for children and adolescents and changing healthcare needs [6].

### Gender incongruence in adolescents and non-binary aspects

To date, there is limited epidemiological data available for gender incongruence in adolescence. In previous school surveys, data ranges from 1.2–1.3% of students identifying as transgender [7, 8] to 2.7% including transgender and gender nonconforming (TGNC) youth [9]. In a nationwide 2021 census in Canada, approximately 0.85% of adolescents aged 15–19 identified as transgender or non-binary [10].

Thereby, self-report data should be interpreted with some caution, given the potentially open and fluid self-discovery processes in adolescents. Individuals with GI often show psychopathological symptoms with a lifetime prevalence for suicide attempts at 14.8% and for non-suicidal self-injurious behavior at 28.2% [11]. There is also evidence for higher rates of eating disorders from transgender college students [12]. Given this context, gender minority stress seems to play a crucial role [13, 14]. The group of non-binary identifying adolescents who do not, or may not exclusively or fully identify with the binary gender categories (“male”/“female”) [15] are particularly at risk of stigmatization and poor mental health outcomes. A recent review indicated that the group of non-binary adolescents experiences elevated levels of depression, anxiety, and suicidal ideation compared to their cisgender and transgender binary peers [16].

According to current medical practice guidelines, adolescents with persisting gender dysphoria can be provided access to gender-affirming hormone treatment (GAHT) with estrogens for birth assigned male and testosterone for birth assigned female transgender persons after careful diagnostic assessment and thoroughly informed consent by adolescents and their legal guardians [17, 18]. Furthermore, gonadotropin-releasing hormone analogues, known as puberty blockers, can be considered as a first stage of medical treatment for adolescents with persisting gender dysphoria, if the progression of irreversible masculinizing or femininizing body changes in either direction shall be postponed for a limited period of time, before a decision for gender affirming hormone treatment can be made [18, 19].

### Maturation processes in the adolescent brain

It is well known that remodeling and developmental processes occur in the brain during adolescence reflected in both a decrease in cortical thickness and increase in white matter [20].

These processes seem to comprise gender-related differences. There is evidence for larger brain surface area in cisgender adolescent boys than for cisgender adolescent girls [21, 22] and evidence for an region-specific earlier maturation in cisgender adolescent girls than boys [23].

Given that adolescence is characterized by substantial neurobiological developmental processes [24, 25] and additionally gender-specific processes in the brain [21, 22], a better insight into neurobiological correlates of persistent GI in adolescence is a promising approach to understand the neurobiological mechanisms underlying GI and especially to improve knowledge for adolescents who may be considered for or undergo gender-affirming hormone treatment.

### Ethical considerations

Due to the progressive irreversible masculinization or feminization in the context of physical maturation, an ethical dilemma arises, since both treatment options (cross-hormonal treatment) and their omission can have severe, in part irreversible consequences [15, 26]. In this ethical consideration, it is essential to account for the volatile developmental and self-discovery processes that are especially pronounced in adolescence, including their neurobiological aspects.

When conducting neurobiological studies e.g. neuroimaging studies on gender-diverse individuals, several important ethical considerations must guide the research: First, human brains are highly diverse. Results must be interpreted within the context of individual variation, rather than reinforcing binary categories. Second, it is essential to avoid implying that brain scans can define or diagnose gender identity. Neuroimaging can never prove or disprove someone’s transgender or gender-diverse identity, and suggesting otherwise risks harmful misunderstandings. Third, there are ethical risks involved, particularly in non-affirming environments. Results could be weaponized to discredit a person’s gender identity, especially for adolescence. Researchers must be mindful of this potential misuse and address it clearly in their work. Fourth, findings should always emphasize the diversity of brain traits across all populations, and it should clearly be spelled out that no one’s identity can be validated or invalidated by brain scans.

Despite the ethically complex situation, neuroimaging remains important for research in this context by contributing to model building and deepening our understanding of how gender diversity may have neurobiological correlates. Some studies have suggested potential overlaps with aspects of neurodiversity; however, this relationship remains debated and should be interpreted with caution [27]. Additionally, it helps to address the complexity of hormonal interventions in adolescence by providing insights into the multiple facets of brain maturation in this context. Additionally, knowledge about hormonal interventions can be improved through research in this field.

### Aim of the review

The objective of this review is to present an overview about the current state of the literature regarding brain magnetic resonance imaging (MRI) findings in adolescents and young adults < 22 years of age with GD/GI also including non-binary identifying adolescents and young adults. The aim is to (a) focus especially on sample characteristics, (b) regard the status of possible hormonal treatment such as cross sex hormones and/or puberty blockers and (c) consider especially non-binary aspects. The aim is also to discuss ethical considerations that arise in the context of investigating neurobiological phenomena in adolescents with GD/GI. Therefore, previous studies are also examined for the research ethics considerations discussed in the “introduction” or “discussion” sections.

## Material and methods

### Search strategy

A systematic literature review was conducted through an electronic search of PubMed^®^ and APA PsycInfo^®^. We used “gender dysphoria”,”gender incongruence “, “transgender”,”gender identity “, “non-binary”, “MRI” and “adolescents” as keywords. We also used the term “transsexualism” to include literature published before 2018.^1^ The protocol was pre-registered (INPLASY2023110074; Date of registration: 11/19/2023). Composition of the review follows the guidelines of PRISMA-ScR (Preferred Reporting Items for Systematic reviews and Meta-Analyses extension for Scoping Reviews) [28].

As a pre-defined search strategy the following key words were used to address the method of interest for reviewing ("MRI" OR "magnetic resonance imaging" OR "imaging" OR "Brain" or "fmri" or "DTI") AND ("adolescents" OR "youth" OR "adolescent"). Please see Supplement 1 for detailed search strategy used in PubMed ®.

### Eligibility criteria

Inclusion criteria were defined as following prior to the review process:

Subjects:

- Female to male transgender individuals (FTM), assigned female at birth (AFAB)
- Male to female transgender individuals (MTF), assigned male at birth (AMAB)
- Non-binary identifying individuals

Methodic criteria

- Human research paper
- English language paper
- Methods: Brain Magnetic Resonance Imaging (MRI)

Exlusion criteria/Papers were rejected according to the following criteria:

- Subjects: adults (> 22 years)
- no original data or case studies or selected case series

There was no restriction regarding the publication date. Citations of the articles found through the described procedure were hand-searched for additional fitting research articles. Our last search was conducted on Oktober 20, 2024.

### Strategy of data synthesis

One reviewer reviewed titles and abstracts found by the given search strategy. Level of full text review ensured correct decision (exclusion or inclusion) when any discrepancies occurred. Data were extracted from the original articles into a summary table. Extraction of the information was double-checked by a different reviewer.

## Main text

**Fig. 1:**
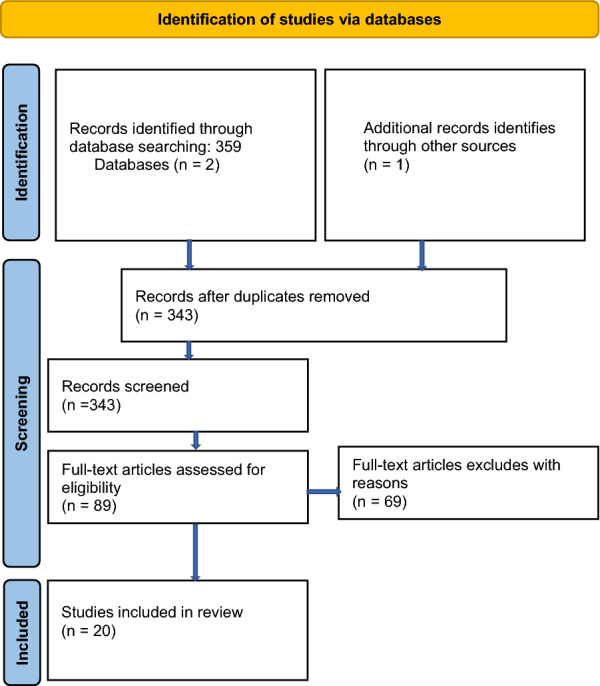
Prisma flow chart diagram (adapted by [29])

Initially, a total of 360 articles were found. After removing duplicates, non-human-research papers and articles not meeting the described inclusion criteria, 20 articles were finally included in the review (see Fig. 1 for detailed strategy).

In a study by Uribe and colleagues, female-to-male transgender persons showed an average age of 22 years. Despite the inclusion criterion of age < 22, the study was included because the subgroup of male-to-female individuals fell within the inclusion criteria (mean age = 20.0) [30].

### Sample characteristics and diagnostic aspects

Regarding diagnostic aspects, with some exception, diagnoses have been confirmed in a center of expertise regarding Diagnostic and Statistical Manual of mental disorders (DSM) criteria (DSM-5 or DSM-IV-TR). As two studies included in the review were embedded in a large population-based-study (Generation R, Rotterdam, The Netherlands; ABCD study, USA), gender diversity was surveyed by a self-rating or by parental report [31, 32].

### Explicit ethical considerations in former studies

Most studies did not actively present ethical aspects and considerations in the introduction and discussion part related to the possible consequences of the findings. In the work by Dhamala and colleagues, ethical considerations are taken into account actively with e.g. stating to consider subjective self-experience and avoid simplistic models [31]. Additionally, the authors explicitly highlight the risk of potential misinterpretation of the results [31]. Non-binary identity was mostly not taken into account. Non-binary identity was explicitly addressed in N = 5 studies [31–35]. In the study by Staphorsius and colleagues, ethical aspects regarding the usage of puberty suppression are discussed [36].

Summing up, in most of the reviewed studies, ethical aspects regarding the potential of.

misinterpretation of findings were not explicitly stated.

### MRI modality

The studies included (N = 20) conducted the following neuroimaging techniques: Structural MRI, Diffusion tensor Imaging (DTI) and functional MRI (fMRI, rs-fMRI). The majority of the studies performed fMRT or functional network analysis (N = 14). Other studies addressed structural imaging (T1) or DTI (see Table 2).

### Longitudinal designs vs. cross-sectional designs

N = 17 studies were limited by cross-sectional designs. Studies performed by Beking et al., Burke et al. (2016) and Nota et al. (2017a) showed a longitudinal design [37–39]. All three studies investigated possible effects of gender affirming hormonal therapy (GAHT). The study of Beking and colleagues performed the second measurement on average 9.8 month after the start of gender affirming hormone therapy (GAHT) in a group of FTM transgender persons (and in a control group of both cisgender adolescent boys and girls) [37]. In the study design of Burke and colleagues, the second measurement took place 10 month after GAHT in FTM transgender persons and in line with Beking and colleagues in a control group of both cisgender adolescent boys and girls [38]. In the study of Nota and colleagues (2017), FTM (female to male) transgender persons were scanned before and 4 month after start of GAHT [39].

To our knowledge, no longitudinal MRI studies have explored the long-term effects of GAHT in adolescent MTF transgender individuals. Moreover, the possible effects of puberty suppression have also not been examined in any longitudinal MRI studies.

### Functional neuroimaging

#### Mental rotation task

In a publication from 2016, Burke and colleagues conducted a mental rotation task, a working memory task with well-documented gender-related performance differences [40, 41]. Using a longitudinal design, the authors showed that FTM transgender persons (before start of GAHT) differed in frontal brain activation from the cis adolescent girls. Interestingly, after 10 months of testosterone treatment, brain activation was increased in areas involved in mental rotation [38].

#### Face matching task

In a cross-sectional study by Grannis and colleagues, FTM receiving Gender affirming hormonal therapy were compared with a group of FTM which never had gender affirming hormonal therapy [33]. Those receiving GAHT exhibited stronger connectivity within the amygdala-prefrontal cortex circuit during a face-processing task compared to those without GAHT. In a subsequent study expanding on these findings, the authors consistently observed stronger connectivity within the amygdala-prefrontal cortex circuit in the FTM group receiving GAHT compared to individuals not receiving GAHT [34].

In a longitudinal design, Beking and colleagues demonstrated a shift of the lateralization index in the face-matching-task in FTM transgender persons towards the right amygdala after testosterone treatment. Furthermore, a significant correlation of the cumulative dose of testosterone treatment with the amygdala lateralization was shown [37].

#### Olfactory stimulation and hypothalamic response

Burke and colleagues were able to show in a fMRI task performed during olfactory stimulation with androstadienone that both adolescent FTM and MTF transgender persons responded not like birth-assigned sex but the gender they identify with [42].

#### Auditory perception task

In a work by Morningstar and colleagues, a group of FTM receiving GAHT and a group of FTM without GAHT were investigated. During fMRI, subjects listened to both angry and happy voices of caregivers and unknown teenagers. For anterior cingulate cortex (ACC), the group receiving GAHT showed blunted neural response to caregivers’ angry voices. Additionally, they showed a heightened response to unknown teenage angry voice with a reverse pattern for the FTM group without hormonal intervention suggesting that testosterone is associated with specific changes in neural processing related to voice recognition and social stimuli [43].

#### Verbal flucency task

In an investigation of regional brain activation during verbal fluency task, no significant differences between FTM, MTF, or cis girls or boys was found [44].

#### Tower of London test (ToL)

Investigating FTM receiving puberty blockade in comparison to youth without puberty blockade, there were no significant differences on ToL performance scores between a group of MTF adolescents undergoing puberty suppression with a group of FTM adolescents not receiving puberty suppression, but significantly lower accuracy scores of the MTF adolescents undergoing puberty suppression compared to both MTF with no hormonal intervention and control group. Thereby, the ToL Test assesses executive functioning [36].

#### Functional connectivity

A study by Nota et al. (2017a) [39] showed that there was no significant difference in functional connectivity among investigated transgender groups. There was no significant difference from the investigated cisgender groups when comparing the investigated FTM groups (both with or without GAHT) [45]. The study was performed with a longitudinal design. After 4 month of treatment with GAHT, there was no difference on functional connectivity [45].

In a study from 2017b, [45] Nota and colleagues were able to demonstrate stronger functional connectivity in the right cerebellum for adolescent MTF compared to adolescent FTM, cis boys and girls. Most interestingly, for cis girls and boys the authors demonstrated differences in the right supplementary motor area and in the right posterior cingulate gyrus. Adolescent MTF showed patterns of FC similar to the gender they indentify with, and adolescent FTM showed a FC pattern similar to their gender they identify with (limited to within the SMN-II) [45]. For detailed results, see Table 1**.**

**Table 1:** Studies performing functional neuroimaging

Authors, year	Technique	Subjects	Control group	Major findings
Beking, 2020 [37]	fMRI: face matching task *longitudinal* *design*	FTM with puberty suppression: N = 21 before start of hormonal transition (MA = 16.1 ys) FTM: N = 21 after starting testosterone treatment (after 9.8 months)	cis girls: N = 20 (MA = 16.4 ys) After 6 M: N = 19 cis boys: N = 19 (MA = 15.9 ys) After 6 M: N = 17	Shift of the lateralization index in FTM transgender persons towards the right amygdala after testosterone treatment significant correlation of the cumulative dose of testosterone treatment
Burke, 2014 [42]	fMRI hypothalamic activations during olfactory stimulation	Pre-pubertal FTM: N = 17 (MA = 9.6 ys) MTF: N = 19 (MA = 10.4 ys) Adolescents with puberty suppression FTM: N = 21 (MA = 16.1 ys) MTF: N = 17 (MA = 15.3 ys)	Pre-pubertal cis girls: N = 19 (MA = 9.7 ys) cis boys: N = 20 (MA = 9.5 ys) Adolescents cis girls: N = 21 (MA = 16.3 ys) cis boys: N = 20 (MA = 15.9 ys)	Both adolescent FTM and MTF responded like gender they identify with pre-pubertal FTM showed neither a typically male nor female hypothalamic activation pattern/ pre-pubertal MTF showed hypothalamic activations that are similar to control boys
Burke, 2016 [38]	fMRI mental rotation task *longitudinal* *design*	FTM: N = 21 (MA = 16.1 ys) FTM (GAHT+): N = 21 (M = 10 months)	cis girls: N = 21 (MA = 16.3 ys) After 6 M: N = 20 cis boys: N = 20 (MA = 15.9 ys) After 6 M: N = 16	FTM before start of GAHT differed significantly in frontal brain activation from the cis girls after 10 months of testosterone treatment: increases in brain activation in areas involved in mental rotation
Dhamala, 2024 [31]	rs-fMRI investigating functional connectivity (FC)	Pre-pubertal FTM: N = 130 (MA = 10.1 ys) MTF: N = 144 (MA = 10.4 ys)	cis girls: N = 2171 (M = 10.2 ys) cis boys: N = 2312 (MA = 10.3 ys)	Functional brain networks are significantly associated with both sex assigned at birth and gender identified with, specific brain network patterns differentiating between cisgender and transgender children (default mode network (DMN) and salience network)
Grannis, 2021 [33]	fMRI face processing task	FTM receiving GAHT(+): N = 19 (MA = 17.0 ys) FTM without hormonal treatment (GAHT−): N = 23 (MA = 15.8 ys)	No control group, comparison between untreated and treated group	FTM (GAHT +): stronger connectivity within the amygdala-prefrontal cortex circuit compared to FTM (GAHT-). Anxiety symptoms moderated by amygdala-prefrontal cortex connectivity differences between the groups
Grannis, 2023 [34] Expansion of [33]	fMRI face processing task	FTM receiving GAHT (+): N = 29 (MA = 16.3 ys) FTM without hormonal treatment (GAHT−): N = 16 (MA = 15.8 ys) MTF receiving GAHT (+): N = 24 (MA = 16.5 ys) MTF without hormonal treatment (GAHT−): N = 12 (MA = 16.1 ys)	cis girls: N = 38 (MA = 16.4 ys) cis boys: N = 40 (MA = 16.2 ys)	FTM (GAHT +) vs. (GAHT-): stronger connectivity within the amygdala-prefrontal cortex circuit. Severity of anxiety and depression was significantly lower; trend of lower suicidality and less distress with body features. Group differences on depression and suicidality directly associated with body image dissatisfaction; anxiety symptoms were moderated by amygdala-prefrontal cortex connectivity differences between groups
Morningstar, 2023 [43]	fMRI Auditory perception task	FTM receiving GAHT+ : N = 19 (MA = 16.2 ys) FTM without hormonal treatment (GAHT−): N = 25 (MA = 15.3 ys)	No control group, comparison between untreated and treated group	GAHT+ youth showed blunted neural response to caregivers’ angry voices; heightened response to unfamiliar teenage angry voices in the anterior cingulate cortex. Reversed pattern in GAHT- group
Nota, 2017a [39]	rs-fMRI investigating functional connectivity (FC) *longitudinal* *design*	FTM without GAHT*: N = 22 (MA = 21.0 ys) After 4 months of GAHT: N = 21 MTF without GAHT*: N = 14 (MA = 21.0 ys) After 4 months of GAHT: N = 13 *partly gonadal supression	cis girls: N = 20 (MA = 23 ys) cis boys: N = 17 (MA = 28 ys)	Within the DMN, SN, and left WMN similar FC patterns were found across groups Cis boys vs. girls: significantly greater FC in the right caudate nucleus within the right WMN no difference in FC among the transgender groups; not significant difference from either of the cisgender groups no effect of GAHT on FC
Nota, 2017b [45]	rs-fMRI investigating functional connectivity (FC)	Pre-pubertal FTM: N = 13 (MA = 9.7 ys) MTF: N = 18 (MA = 10.5 ys) Adolescents FTM: N = 21 (MA = 16.1 ys) MTF: N = 19 (MA = 15.4 ys)	Pre-pubertal cis girls: N = 18 (MA = 9.6 ys) cis boys: N = 21 (MA = 9.4 ys) Adolescents cis girls: N = 21 (MA = 16.3 ys) cis boys: N = 20 (MA = 15.9 ys)	Adolescent MTF vs. Adolescent FTM, cis boys and girls: stronger FC in the right cerebellum cis girls vs. boys: Sex differences in the right supplementary motor area within one of the two SMNs (SMN-II; girls > boys) and the right posterior cingulate gyrus within the posterior DMN (boys > girls). Adolescent MTF: FC patterns similar to the gender they identify with (female). Adolescent FTM: showed a FC pattern similar to gender identified with (male), but within the SMN-II only prepubertal children: no group differences in FC
Skorska, 2022 [46]	rs-fMRI	FTM: N = 17 (MA = 15.4 ys)	cis girls: N = 17 (MA = 16 ys) cis boys: N = 15 (MA = 16 ys)	FA differed among cis boys, cis girls and FTM largest difference between cis boys and FTM brain functional organization of FTM more similar to cisgender girls than cisgender boys
Soleman, 2013 [44]	fMRI Verbal fluency test	FTM: N = 14 (MA = 14.6 ys) MTF: N = 8 (MA = 14.3 ys)	cis girls: N = 26 (MA = 14.4 ys) cis boys: N = 25 (MA = 14.7 ys)	Cis boys showed more activation in the right Rolandic operculum compared to girls. FTM. vs. MTF: No significant differences in brain activity (sub-threshold activation was found in the right Rolandic operculum trendwise increase in activation < cis girls < FTM < MTF < cis boys
Staphorsius, 2015 [36]	fMRI, ToL Test	FTM receiving puberty suppression: N = 12 (MA = 16.1 ys) FTM without treatment: N = 10 (MA = 15.4 ys) MTF puberty suppression: N = 8 (MA = 15.4 ys) MTF without treatment: N = 10 (MA = 14.6 ys)	cis girls: N = 24 (MA = 14.4 ys) cis boys: N = 21 (MA = 14.9 ys)	FTM (ps +) vs. FTM (ps-): no significant differences on ToL performance scores. MTF (ps +) vs. MTF (ps-): no significant differences on ToL performance scores. MTF (ps +): significantly lower accuracy scores compared to MTFp-, and control groups. MTFps + vs. FTMps + Greater activation in the other ROIs (left DLPFC and bilateral RLPFC)
Strang, 2023 [47]	rs-fMRI	Transgender individuals (non-autistic): N = 16 (MA = 17.7 ys) Transgender individuals (sub-clinical autistic): N = 14 (MA = 18.1 ys) Transgender individuals (autistic): N = 15 (MA = 18.0 ys)	No control group, comparison between untreated and treated group	Subclinically autistic group differed vs. non-autistic and full-criteria autistic groups: DMN hub connectivity to ventral attention and sensorimotor networks, hyperconnectivity between DMN hub and dorsal attention network
Uribe, 2020 [30]	T2 weighted (Functional connectivity)	FTM: N = 29 (MA = 22.0 ys) MTF: N = 17 (MA = 20.0 ys)	cis women: N = 22 (MA = 19.0 ys) cis man: N = 19 (MA = 22.0 ys)	MTF, FTM cisgender women vs. cisgender men: decreased connectivity in superior parietal regions, as part of the salience (SN) and the executive control (ECN) networks. FTM vs. cisgender men: weaker connectivity compared with cisgender men between intra-SN regions and weaker inter-network connectivity between regions of the SN, the default mode network (DMN), the ECN and the sensorimotor network. MTF vs. cisgender man: lower small-worldness, modularity and clustering coefficient than cisgender men no differences among FTM, MTF and ciswomen

*cis*   *gender identity that aligns with birth-assigned sex *

*MTF*   *male* *to* *female, FTM*   *female* *to* *male, fMRI* *functional MRI, MA*   *Mean Age, GAHT*±     *Group receiving/ not receiving gender affirming hormonal therapy, rs-fMRI*   *resting state fMRI, FC*   *Functional connectivity, DMN*   *Default mode network, SN*   *Salience Network, WMN*   *Working memory network, SMN*   *sensorimotor networks, ps*±    *receiving/not receiving puberty suppression, ROI*   *region of interest, DLPFC*   *Dorsolateral prefrontal cortex, RLPFC*   *Rostrolateral prefrontal cortex, ECN*   *Executive control network*

In a work by Skorska and colleagues the authors showed that functional connectivity differed among cis boys, cis girls and FTM with the largest difference between FTM and cis boys [46]. Uribe and colleagues showed decreased connectivity in superior parietal regions between MTF, FTM, cisgender women and cisgender men [30]. Most interestingly, the authors also showed a weaker connectivity in FTM compared with cisgender men between intra-salience-network regions and weaker inter-network connectivity between regions of the salience network: default mode network (DMN), executive control network and the sensorimotor network. Regarding network metrics, MTF transgender persons showed a lower small-worldness, modularity and clustering coefficient than cisgender men [30].

In a large cohort, specific brain network patterns in prepubertal transgender children showed significant differences in the default mode network (DMN) and salience network which are related to self-perception, identity, and social processing [31].

### T1-weighted (grey matter morphometry) and diffusion tensor imaging (DTI)

Structural neuroimaging (n = 4) [32, 48–50] and DTI (n = 1) [35] studies used cross-sectional designs, mostly including both FTM and MTF individuals. However, Skorska et al. (2021, 2023) focused only on FTM individuals [49, 50].

### Whole brain analyses, vertexwise analyses and region of interest analyses (ROI)

Whole-brain analyses revealed that FTM and MTF adolescents differed from their gender-identity group but not from their sex-assigned-at-birth group. In a multivariate pattern recognition analyses, it was possible to discriminate transgender persons better from subjects sharing their gender identity than their sex assigned at birth [48]. A population-based study found no global volumetric differences in gender-diverse adolescents [32].

In vertexwise analyses in adolescents assigned male at birth, thicker cortices in the left inferior temporal gyrus were found among youths who reported gender diversity compared to adolescents who reported cisgender identity. Between the group of identifying as gender diverse and surface area in vertexwise analyses, no associations were found [32].

In a study by Skorska and colleagues (2021) [49] it was shown that cisgender boys had higher surface area (SA) compared to cisgender girls and FTM transgender persons [49]. Most interestingly also sexual orientation was considered. It could be shown that shorter T1, a marker for denser structures resulting from high concentrations of macromolecules correlated with older age and more pronounced gynephilia in both cisgender boys and FTM transgender persons [49]. In summary, cortical morphometry reflected in mainly SA) was related to sex assigned at birth, but not the gender they identify with [49].

A study by Skorska and colleagues (2023) [50] demonstrated for both FTM transgender persons and cisgender boys that in several brain regions, such as left frontal, parietal, and temporal regions a combination of shorter T1 relaxation time and faster mean diffusivity was linked with higher age and larger levels of gynephilia [50] For detailed results, see Table 2.

**Table 2:** Studies performing structural neuroimaging

Authors, year	Technique	Subjects (GD)	Control group	Major findings
Hoekzema, 2015 [48]	T1 (3 T)	FTM*: N = 54 (MA = 16.2 ys) *(GAHT/ps−: n = 17, ps: n = 16, GAHT+ : n = 21) MTF*: N = 37 (MA = 16.5 ys) *(GAHT/ps−: n = 11, ps: n = 14, GAHT+ : n = 12)	cis girls: N = 52 (MA = 16.3 ys) cis boys: N = 44 (MA = 15.9 ys)	Whole-brain level: GD groups differed from subjects sharing gender identity but not from sex assigned at birth multivariate pattern recognition analyses: GD groups: more accurately be discrimination from subjects sharing gender identity than sex assigned at birth. ROI-analyses: FTM vs. cisgender girls: less GM volume in right cerebellum and more volume in the medial frontal cortex. MTF vs. cisgender boys: less GM volume in the bilateral cerebellum and hypothalamus
Skorska, 2021 [49]	T1, (3 T) surface area (SA) cortical thickness (CT) T1 relaxion time	FTM without hormonal treatment: N = 16 (MA = 16.1 ys)	cis girls: N = 17 (MA = 16 ys) cis boys: N = 14 (MA = 15.4 ys)	Cis gender boys vs. cis girls and FTM: larger SA. Cortical morphometry (SA) related to sex assigned at birth but not gender they identify with
Skorska, 2023 [50]	T1 relaxometry	FTM: N = 15 (MA = 16.1 ys)	cis girls: N = 17 (MA = 16 ys) cis boys: N = 14 (MA = 15.4 ys)	Multivariate analyses: cortical T1 relaxation time showed weak statistically significant positive association with MD across the cortex in several left frontal, parietal, and temporal regions, combination of shorter T1 relaxation time and faster MD was associated with older age and greater gynephilia in FTM and cisgender boys and with stronger attractions in cisgender boys only for these cortical regions in these groups, older age, gynephilia, and stronger attractions (cisgender boys only) were associated with macromolecule-rich tissue in which water movement was freer
Thurston, 2024 [53]	DTI, (3 T) TBSS	FTM without hormonal treatment: N = 17 (MA = 15.5 ys)	cis girls: N = 17 (MA = 15.5 ys) cis boys: N = 14 (MA = 15.4 ys)	Significant group differences in axial diffusivity (AD) and its correlation with T1 relaxation times. Stronger correlations between T1, AD and serum estradiol, pubertal development, and sexual attraction in cisgender boys compared to cisgender girls and transgender boys
Van Heesewijk, 2022 [35]	DTI, (3 T)	Children: MTF: n = 20 (MA = 10.4) Children: FTM: n = 15 (MA = 9.6) Adolescents: MTF, ps+: n = 20 (M_age_ = 15.4) FTM, ps+: n = 21 (MA = 16.1)	Children: cisgender boys n = 18 (MA = 9.6) Children: cisgender girls n = 20 (MA = 9.7) Adolescents: cisgender boys n = 20 (MA = 15.9) cisgender girls n = 21 (MA = 16.3)	Lower FA in the bilateral inferior fronto-occipital fasciculus (IFOF), forceps major and corpus callosum. Average FA values of the right IFOF correlates negatively with cumulative dosis of puberty suppression received
Xerxa, 2023 [32]	MRI (3 T) T1	Gender non-conforming: N = 96	N = 2096	Global brain volumetric measures: no difference between adolescents reporting gender diversity vs. not reporting whole-brain, vertexwise analyses: among adolescents assigned male at birth reporting gender diversity vs. not reporting: thicker cortices in the left inferior temporal gyrus. No associations between gender diversity and surface area (vertexwise analyses)

*MTF*   *male to female, FTM*   *female to male, cis*   *gender identity that aligns with birth-assigned sex, MA* *Mean age, GD*   *Gender dysphoria, ROI*   *Region of interest, AD*   *Axial Diffusity, IFOF*   *inferior fronto-occipital fasciculus, FA*   *Fractional Anisotropy, SA*   *Surface Area, CT*   *Cortical Thickness, MA*   *Mean age, T*   *Tesla, ps* ±    *receiving/not receiving puberty suppression, DTI*   *Diffusion Tensor Imaging, GAHT* ±   *Group receiving/ not receiving gender affirming hormonal therapy*

Regarding region of Interest (ROI)-analyses, FTM individuals showed less grey matter (GM) volume in right cerebellum and more volume in the medial frontal cortex compared to cisgender girls in a study by Hoekzema and colleagues [48]. In MTF individuals, the authors demonstrated less GM volume in the bilateral cerebellum and hypothalamus compared to cisgender boys [48].

Summing up, while some aspects of brain morphology in transgender adolescents seem to be strongly influenced by the sex assigned at birth, other differentiated characteristics may align more with individuals sharing the same gender identity.

### Diffusion tensor imaging

Diffusion Tensor Imaging (DTI) is a method to investigate changes of white matter in vivo [51]. As summarized in Table 2, Van Heesewijk investigated fractional anisotropy (FA) a MRI measure that characterizes white matter microstructure in adolescents with gender dysphoria [35]. Most interestingly, the bilateral inferior fronto-occipital fasciculus (IFOF) showed lower FA, which has also been described in transgender adults [52].

### Associated psychopathology and diagnoses

In the study by Grannis and colleagues (2021), [33] generalized anxiety, social anxiety, depression, suicidality and body image dissatisfaction were assessed in both treated FTM adolescents (GAHT+ : n = 19) and in non-treated FTM adolescents (GAHT–: n = 23). The treated group showed lower severity of anxiety and depression, less body image distress, and a trend toward reduced suicidality compared to the untreated group [33]. These results were stable in an expansion of the reviewed work from 2023 [34].

In the study by Strang and colleagues, distinct patterns of hyperconnectivity in the default mode network (DMN), particularly in connections with visual, motor, and attention networks were found to differ across non-autistic, subclinically autistic, and fully autistic transgender youth, with subclinical autism showing unique connectivity characteristics [47].

## Conclusion

In summary, in this scoping review we identified n = 20 studies published between 2013 and 2024. Studies mostly investigated functional imaging aspects.

In a recent review regarding findings in adult transgender person, it is postulated that there is evidence for a gender-identity-associated brain organization (brain gender) [54]. Regarding adolescence, we summarize, that there is also some preliminary evidence for “sex-atypical”, gender-identity-associated brain organization [38, 42, 48] in adolescent transgender persons as also stated in a recent review [55]. Thereby, findings indicate some evidence for a brain phenotype that is not simply shifted to the male or female spectrum [55]. These findings should be interpreted with caution, given that research should never imply that transgender identities or other identities can be proven or disproven by neuroanatomy, and should always be seen in the individual context of self-experience. Previous research could place greater emphasis on this aspect to prevent the unethical instrumentalization of results.

Nearly all reviewed studies are limited by small sample sizes. Particularly with regard to the developing adolescent brain and its plasticity, solid neuroimaging data helping to understand the neurobiological mechanisms associated with gender diversity are still lacking.

Furthermore, there is a lack of longitudinal data considering male to female transgender individuals undergoing gender affirming hormone treatment, whereas some longitudinal data is published for female to male transgender individuals [37–39]. To date there are neither longitudinal data available for puberty suppression. Only few studies to date address non-binary-identity [32–34].

It should be pointed out, that in n = 18 studies, diagnosis took place in specialized treatment centers so that one might assume a high diagnostic standard according to current guidelines [17].

### Strength and limitations

The focus on transgender adolescents, extending up to 22 years of age,—shedding the light on the relevant period of adolescence in the context of brain maturation—seems to be the main strength of our review. Our review has also a few limitations. To ensure feasibility, we focused solely on English language literature. The limited number of studies suitable for reviewing with relatively small cohorts has to be pointed out as a relevant limitation reducing the generalizability. Most available studies relied on cross-sectional designs, which restricts conclusions regarding developmental trajectories. Furthermore, heterogeneity in imaging protocols, analytical methods, and diagnostic/sample definitions complicates direct comparison across studies. Finally, while we highlight ethical considerations, our review cannot capture the perspectives of transgender and non-binary individuals themselves, which remains an important aspect for future research.

### Future directions

In light of the findings of this scoping review, future study designs should be guided by the specific gaps identified here. While functional neuroimaging was most frequently applied (see Table 1), studies addressing structural imaging and diffusion tensor imaging were relatively scarce (see Table 2), suggesting that multimodal designs integrating functional, structural, and connectivity measures would be important approaches. Although some longitudinal data exist for FTM adolescents undergoing GAHT [37–39], no longitudinal MRI studies have yet been conducted in MTF adolescents, in the context of puberty suppression, or in non-binary youth. Addressing these gaps will be critical for understanding developmental trajectories across the full spectrum of gender-diverse adolescents. Given that most studies did not include non-binary adolescents, there is a need for more inclusive sampling strategies in future research. Future studies may benefit from including dimensional measures of gender experience [56], as it is important to assess how gender is experienced even in control groups. Finally, the frequent reliance on small sample sizes in single-center studies indicates that multicenter collaborations and data-sharing initiatives will be essential to achieve adequately powered analyses, as has already been successfully demonstrated in adult research [57].

Especially longitudinal designs with the possibility of follow-up measurements focusing on both MTF but also FTM transgender persons, as well as non-binary individuals, seem to be highly advisable in order to better understand the neurobiological processes in GI/GD which might lead to an improvement of the theoretical understanding of the neurobiological mechanisms associated with gender incongruence. It can be stated in line with a recent review [55] that further research is urgently needed focusing on bio-psycho-social maturation processes.

Based on the paradigm shift of de-pathologization and de-stigmatization of gender-diverse identities, there are also ethical aspects that should be considered when conducting studies. As this scoping review showed, only very few studies included adolescents identifying as non-binary.

In future research designs ethical aspects should be carefully taken into account, particularly—with appropriate sensitivity to avoid repudidation or stigmatization. Studies should not only use gender-sensitive language and avoiding ascriptions of gender-diverse persons as being abnormal, but should incorporate the perspective of gender-diverse persons in posing adequate research questions as well as interpreting data through adequate participation. In this effort, research objectives aiming at a better understanding of neurobiological mechanisms associated with gender diversity should not be mistaken in any simplistic model, in which the unique and complex experience of a gender-diverse person is reduced to its associated neurobiology or neurodiversity.

Future research projects should consider implementing an Ethical Advisory Board with at least one experienced GI healthcare professional and one trans person. This board would offer crucial guidance on study design and result interpretation, ensuring the research remains ethical, inclusive, and responsive to diverse perspectives.

## Supplementary Information

Supplementary Material 1.

## Data Availability

No datasets were generated or analysed during the current study.

## Abbreviations

ACC: Anterior cingulate cortex
AD: Axial diffusivity
AFAB: Assigned female at birth
AMAB: Assigned male at birth
DSM: Diagnostic and Statistical Manual of mental disorders
DTI: Diffusion tensor imaging
GAHT: Gender-affirming hormone treatment
GAHT+: Group receiving Gender-affirming hormone treatment
GAHT: Group not receiving Gender-affirming hormone treatment
GD: Gender dysphoria
GI: Gender incongruence
FA: Fractional anisotropy
FC: Functional connectivity
FTM: Female-to-Male
fMRT: Functional Magnetic resonance imaging
ICD-11: International classification of diseases, 11th revision
MTF: Male-to-Female
MRI: Magnetic resonance imaging
ps+: Group with puberty supression
ps−: Group without puberty suppression
ROI: Region of interest
TGNC: Transgender and gender nonconforming
ToL: Tower of London task

## Footnotes

1 In ICD-11 GI is no longer listed as a psychiatric diagnosis as in former ICD-10 (“transsexualism”), but rather a condition related to sexual health.
