# Supplementary material for: Neuroimaging insights into transgender and gender nonconfirming youth: a scoping review
Source: Child Adolesc Psychiatry Ment Health. 2025 Nov 26;19:135. doi: 10.1186/s13034-025-00987-1 (PMC12670818; doi:10.1186/s13034-025-00987-1)
Supplement: Supplementary file 1 — Supplementary Material 1. [file 13034_2025_987_MOESM1_ESM.docx]

Supplement 1: Detailed search strategy

Search strategy for pubmed ®

("gender dysphoria" OR "gender incongruence" OR "gender identity" OR "non-binary" OR "transsexualism" or "transgender") AND ("MRI" OR "magnetic resonance imaging" OR "imaging" OR "Brain" or "fmri" or "DTI") AND ("adolescents" OR "youth" OR "adolescent")

Key words:

Gender dysphoria Gender incongruence Gender identity Non-binary Transsexualism Transgender

MRI

Magnetic resonance imaging
Brain

fMRI

DTI

Adolescents

Youth

adolescent
